# A pharmacist-delivered smoking cessation program in Qatar: an exploration of pharmacists’ and patients’ perspectives of the program

**DOI:** 10.1007/s11096-021-01286-3

**Published:** 2021-06-02

**Authors:** Maguy Saffouh El Hajj, Saba Abdal Salam Sheikh Ali, Ahmed Awaisu, Rana Saleh, Nadir Kheir, Rula Shami

**Affiliations:** 1 Department of Clinical Pharmacy and Practice, College of Pharmacy, QU Health Qatar University, 2713 Doha, Qatar; 2 Pharmacy Department, Hamad Medical Corporation, Doha, Qatar; 3 College of Pharmacy, Ajman University, Ajman, United Arab Emirates; 4 College of Health Sciences, QU Health Qatar University, 2713 Doha, Qatar

**Keywords:** Pharmacist, Qualitative research, Smoking cessation, Tobacco cessation

## Abstract

*Background* Tobacco use is one of the major causes of morbidity and mortality. An intensive pharmacist-delivered smoking cessation program was implemented in eight primary care pharmacies in Qatar. *Objective* This study aimed to qualitatively explore the perspectives of pharmacists and patients regarding their experiences in the program and their recommendations for improving it. *Setting* Primary care in Doha, Qatar. *Method* This study used a qualitative case study approach with semi-structured interviews of a sample of patients and pharmacists who participated in the program. Interviews were conducted between October 2016 and June 2017, were audio-recorded and transcribed verbatim. A thematic approach for data analysis was used. *Main outcome measures* Perspectives of pharmacists and patients. *Results* Pharmacists who delivered the program (n = 17) and patients who completed the program’s outcomes assessment (n = 68) were invited through telephone call or email. Eight pharmacists and 22 patients were interviewed. Seven themes emerged: (1) both pharmacists and patients had positive experiences and both considered pharmacists as among the most suitable healthcare providers to provide smoking cessation interventions (2) both pharmacist and patient participants indicated that the program provided successful services (3) pharmacists identified several challenges for implementing the program including difficulty in motivating and in following-up patients, workplace barriers, communication and cultural barriers, (4) both pharmacists and patients perceived several barriers for quitting including lack of motivation to quit or to commit to the plan, high nicotine dependence, stress and personal problems (5) both pharmacists and patients considered several patient-related facilitators for quitting including development of smoking related complications, religious beliefs and external support; (6) use of smoking cessation medications was considered a program-related facilitator for quitting by patients whereas behavioral therapy was perceived to be a facilitator by pharmacists (7) pharmacists and patients proposed strategies for program improvement including enhancing pharmacist training and patient recruitment. *Conclusion* The program was perceived to be beneficial in helping patients quit smoking, and it positively contributed to advancing pharmacist role. The study findings can guide future development of successful pharmacist’ smoking cessation programs in Qatar.

## Impacts of practice

- The study highlighted the important role of pharmacist in smoking cessation in Qatar.
- The study determined the challenges and facilitators for implementation of pharmacist-delivered smoking cessation programs.
- The study identified the positive aspects of the pharmacist-run smoking cessation program and the negative aspects that would need improvement for designing effective and sustainable tobacco cessation intervention programs in the future.
- The study findings can potentially help future pharmacists in Qatar and probably in the Middle East and beyond in planning and executing effective pharmacist delivered smoking cessation programs.

## Introduction

Tobacco use is a primary cause of premature morbidity and mortality and is responsible for the global mortality of over 8 million people annually [1, 2]. Tobacco cessation is associated with several health benefits [3]. However, because of the addictive nature of tobacco use, self-help for tobacco cessation can be very challenging, leading to failure of several self-initiated quit attempts. Provision of tobacco cessation interventions by healthcare practitioners is proven to be more effective than self-help [4].

Pharmacists are considered as one of the most accessible healthcare providers with frequent patient interactions and without the need for an appointment. They have a great opportunity to contribute to health promotion and disease prevention activities including smoking cessation [5]. Evidence has proven that pharmacists can be effective in providing smoking cessation interventions and in assisting patients to quit [6–8].

The prevalence of tobacco use constitutes a major health burden in Qatar. According to the 2013 Global Adult Tobacco Survey (GATS), 12.1 % of adults in Qatar currently smoke tobacco and around 84 % of the current cigarette smokers are daily smokers [9]. Tobacco-related diseases are also highly prevalent in Qatar [10, 11]. Qatar government has implemented several anti-tobacco legislations and programs [12]. Yet tobacco use is still a public health problem. Qatar pharmacists can play a significant role and contribute in combating the tobacco use epidemic in the country.

In 2010, a cross-sectional survey among community pharmacists in Qatar has shown that more than 85 % of pharmacists expressed interest in providing smoking cessation counseling [13]. In light of these results, the first randomized controlled trial to investigate the effectiveness of a pharmacist-delivered smoking cessation program in Qatar was conducted. In this program, 314 smokers were randomized into two groups: intervention and control groups. Participants in the intervention group met with the pharmacists every 2–4 weeks in a four-session structured and intensive smoking cessation program [14]. Participants in the control group received unstructured brief smoking cessation counseling. The smoking abstinence rate at 12 months was higher in the intervention group, although this did not reach statistical significance (23.9 % vs. 16.9 %; *p* = 0.257) [14].

As this was a new program, it is crucial to explore the patients’ and the pharmacists’ experiences in the program in order to identify the positive aspects of the program and those aspects that would require improvement for the purpose of designing effective and sustainable tobacco cessation intervention programs in the future. A number of qualitative studies were conducted in different countries to gauge the perspectives of pharmacists or patients in relation to pharmacist-delivered smoking cessation interventions [15–17]. The findings of these studies were very useful in exploring the strengths and weaknesses of the interventions and in improving them. Nevertheless, the results of these studies cannot be applied to Qatar, a country with a very different and unique healthcare system.

### Aims of the study

This study aimed to explore the perspectives of pharmacists and patients in Qatar regarding their experiences in the pharmacist-delivered smoking cessation program and their recommendations for improving the program.

## Methods

### Study design

A qualitative case study research approach using semi-structured interviews was used to explore the perspectives of pharmacists and patients in Qatar regarding their experiences in the program.[18].

### Study participants and recruitment

 Pharmacists who delivered the smoking cessation program (n = 17) and patients who participated in the intervention group and completed the program’s outcomes assessment (n = 68) were invited to participate in semi-structured interviews. Semi-structured interviews were considered more appropriate than focus groups due to the difficulties entailed with selecting suitable times for an appropriate number of pharmacists and patients and due to the sensitive nature of the topic of smoking cessation [19].

### Interview guide development

For consistent and structured data collection, interviews were conducted using a semi-structured interview guide. The questions were designed based on the program’s objectives and published literature related to pharmacist-delivered smoking cessation programs. To assess its validity, the guide was reviewed by pharmacy practice research professors with expertise in qualitative research and tobacco cessation. The guide was translated to Arabic using International Society for Pharmacoeconomics and Outcomes Research (ISPOR) Principles of Good Practice for the Translation and Cultural Adaptation Process for Patient-Reported Outcomes (PRO) Measures [20].

### Interviews structure

The interviews were conducted in English or Arabic at Qatar University College of Pharmacy between October 2016 and June 2017 in Doha, Qatar. Each Interview was about 60–120 min. Each interview involved one moderator (MH: Associate Professor of pharmacy practice) and one recorder (SS: student researcher). The researchers did not have any established relationships with the participants. At the end of each interview, the research team met to reflect on how the interviews went and to improve future interviews if needed [21].

### Data transcription and analysis

The interviews were audio-recorded and transcribed verbatim. Arabic transcripts were translated to English. The transcripts were compared with the recorder’s notes after each interview by one team member and validated by another member. The final transcripts were also sent to participants in case they have any comments. Qualitative data analysis was conducted along with data collection. This permitted the research team to constantly review the collected data and to design new questions for consequent interviews if needed [22]. The team read the transcripts several times and a list of codes was generated based on recurrent trends and patterns. Thematic analysis was conducted manually. Two study investigators (MH and SS) divided the text into small elements of meaning or words, and individually coded the transcripts into themes and subthemes [22, 23]. Themes and subthemes were compared by different team members. Agreement was obtained through discussions. The results were shared with interviewees through email for any comment. The study was conducted and reported in accordance with the Consolidated Criteria for Reporting Qualitative Studies (COREQ) [24].

## Ethics approval

 The study was approved by the Qatar University Institutional Review Board (reference number: QU-IRB 686-E/16 in 2016) and the Primary Health Care Corporation Research Committee (reference number: 11,113/11 in 2016).

## Results

**Fig. 1:**
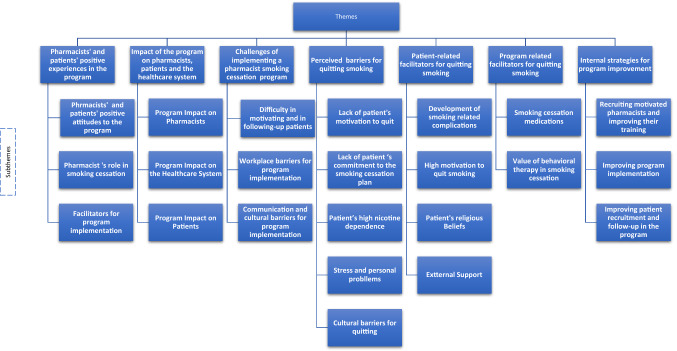
Conceptual diagram of key themes and subthemes for pharmacists’ and patients’ interviews

Thirty one-to-one interviews were conducted; eight interviews with pharmacists and 22 interviews with patients (Table 1). No further interviews were deemed necessary as data saturation was achieved. Seven themes were generated from the interviews (Fig. 1).

**Table 1:** Interview participants

	Nationality	Years in Qatar	Gender
*Patients*			
Pt-01	India	11 years	Male
Pt-02	Egypt	8 years	Male
Pt-03	Egypt	6 years	Male
Pt-04	Egypt	13 years	Male
Pt-05	Egypt	8 years	Male
Pt-06	India	5 years	Male
Pt-07	Egypt	14 years	Male
Pt-08	Egypt	15 years	Male
Pt-09	Philippines	7 years	Male
Pt-10	Philippines	9 years	Male
Pt-11	Not disclosed	5 years	Male
Pt-12	Egypt	6 years	Male
Pt-13	Egypt	13 years	Male
Pt-14	Malaysia	8 years	Male
Pt-15	India	9 years	Male
Pt-16	Philippines	6 years	Male
Pt-17	Tunisia	9 years	Male
Pt-18	Philippines	Not disclosed	Male
Pt-19	Sudan	20 years	Male
Pt-20	Syria	6–7 years	Male
Pt-21	Jordan	Not disclosed	Male
Pt-22	Bangladesh	24 years	Male
*Pharmacists*			
PD1-01	Pakistan	15 years	Male
PD1-02	Sudan	25 years	Male
PD1-03	Egypt	5 years	Male
PD2-01	Sudan	26 years	Male
PD2-02	Sudan	6 years	Male
PD2-03	India	8 years	Male
PD2-04	Jordan	10 years	Male
PD3-01	Jordan	5 years	Female

### Theme 1: Pharmacists’ and patients’ positive experiences in the program

#### Subtheme: Pharmacists’ and patients’ positive attitudes towards the program

Pharmacists and patients generally expressed very positive attitudes towards the pharmacist-led tobacco cessation program. In particular, they expressed happiness and satisfaction in the program’s organization and how patient care was generally provided. The pharmacists indicated that communication with patients was smooth and that approaching patients and initiating smoking cessation discussion with them was overall easy.

“I think it was well-structured, patient profile was well organized, it was very easy to interact with these patients” PD1-02.

#### Subtheme: Pharmacist’s role in smoking cessation

Pharmacists and patients considered the pharmacists as one of the most suitable healthcare providers to provide smoking cessation interventions since they are the most accessible to the public and they have more time to interact with patients as compared to other healthcare providers. They also stated that pharmacists are often considered as medication therapy experts and patients would prefer pharmacists’ interventions over other healthcare providers.

“the pharmacist has more time to interact with me as a patient than other healthcare professionals… the doctor sees over 40 to 50 patients daily he or she may not have the time to see me the pharmacist is there to answer my questions he or she is the expert for medications” P2.

However, some patients criticized certain pharmacists for not being adequately trained and for their limited role to only dispensing medications.

“I don’t think they are trained enough. I think they focus on dispensing the medication only, that’s what my feeling is, you get this feeling that you are going to a hospital or a doctor expecting to be an expert… you know it is his or her field… so especially you’re talking to a pharmacist, you expect that he or she knows about it all but that’s not the case…” P15.

#### Subtheme: Facilitators for program implementation

Pharmacists identified several facilitators for the successful implementation of the program. This included availability of private consultation rooms in some clinics, equipment, and free of charge medications in addition to management support.

“One of the program facilitators was the availability of smoking cessation medications for free, actually, most of the patients when they hear that they will take free patch, or free lozenges, they will come” PD1-02.

### Theme 2: Impact of pharmacist-provided smoking cessation program on pharmacists, patients and the healthcare system

#### Subtheme: Program impact on pharmacists

The pharmacists believed that the program had a positive impact on their profession; they summarized this impact as: improved job satisfaction and career development, and a great opportunity to participate in a research project that would highly serve the society.

“certain patients are still in touch with me, because they are regular patients in our center, when they see me they tell me Dr we stopped smoking after your advice, so it was an achievement for me” PD3-01.

#### Subtheme: Program impact on the healthcare system

Pharmacists mentioned that after hosting the program, different healthcare systems in Qatar started expanding the services that they provide to the community and focused on smoking cessation counseling. They also stated that physicians had positive attitudes towards the program and were cooperative with its activities and requested that the pharmacist run program would continue.

“I found it useful actually, for myself and for my career even for my health center, physicians asked us to continue the program in our pharmacy” PD1-02.

#### Subtheme: Program impact on patients

Both pharmacist and patient participants indicated that the program provided successful smoking cessation services for the patients. They were confident that the program improved the patients’ general health and wellbeing and improved their awareness about the importance of smoking cessation.

“I can consider this program um… as a successful program, because it spread awareness about the smoking risks, even though it did not help the smokers stop smoking completely but it gave them the knowledge of why they should stop smoking. This basic knowledge was lacking,” PD3-01.

### Theme 3: Challenges of implementing a pharmacist-run smoking cessation program

#### Subtheme: Difficulty in motivating and in following-up patients

Pharmacists expressed concerns on and difficulty in initiating discussions with patients who are not motivated to quit and in following-up with patients.

“it’s not much easy. For example, like, we have to motivate patients to join the program and to do the follow-up by calling them, it’s not that easy as we think” PD1-01.

In addition, the participants identified several logistic factors that might lead to loss to follow-up including change in patient’s location, transportation issues, having the clinic in a distant location, pharmacist’s busy schedules, and difficulty in arranging follow-up appointments.

“Because my clinic is so far … I called them several times to remind them about their appointments…some of them told me frankly… I cannot come. it is a very far area… if you want to give me any advice please do so on the phone” PD2-01.

#### Subtheme: Workplace barriers for program implementation

Pharmacists identified several workplace barriers that restricted their ability to effectively implement the program. These included lack of private consultation area for meetings in some clinics, inability of pharmacists to shift between the pharmacy and the clinic, and lack of cooperation from some healthcare professionals. Pharmacists also mentioned lack of an organized referral system from physicians, necessity for an appointment for patients to attend the clinic, and inability of pharmacists to measure the patients’ vital signs.

“Some physicians do not trust the pharmacists to run this program because according to their knowledge which is very traditional… this patient should undergo several investigations and should see the physician first before seeing the pharmacist” PD1-01.

#### Subtheme: Communication and cultural barriers for program implementation

Pharmacists mentioned several communication and cultural barriers. First, pharmacists found it difficult to communicate and counsel patients who cannot understand English or Arabic. Many patients were coming from Asian countries and they had difficulty in communicating in English or Arabic.

Many pharmacists also faced cultural challenges while interacting with female patients as well as with young smokers who are concerned about the social stigma associated with smoking. In fact, many female patients were very shy to approach the pharmacists to speak about smoking cessation freely.

“We had a very small community… there were many young patients… maybe if they enter the health center, they are afraid somebody will see them and will know that they smoke they are embarrassed to smoke in the public they are afraid of what the society would say about them” PD1-03.

### Theme 4: Perceived barriers for quitting smoking

#### Subtheme: Lack of patient’s motivation to quit

Pharmacists and patients alike agreed that despite the fact that smoking is becoming a health burden worldwide, some smokers are still not motivated to quit. They indicated that many patients were not ready to take this step especially the young ones.

“So many of the smokers we have encountered are young smokers who are under peer pressure to smoke and all what they enjoy is smoking with friends… so they don’t want to listen. and with those young smokers we feel like they are not motivated to quit.” PD1-01.

#### Subtheme: Lack of patient‘s commitment to the smoking cessation plan

Pharmacists added that many patients found it difficult to make serious lifestyle changes by committing to their smoking cessation plans although they were willing to quit.

“Some intervention patients did not attend the follow-up sessions, despite being motivated they could not commit to the smoking cessation plan by making changes to the recommended lifestyle changes ” PD3-01.

#### Subtheme: Patient’s high nicotine dependence

Pharmacist and patient interviewees indicated that many patients were heavy smokers and highly dependent on nicotine; therefore, they required stronger medical interventions than nicotine replacement therapy.

“some patients were very addicted and had high nicotine dependence they tried to quit before three or four times and they failed in quitting I believe they needed stronger medications” PD2-01.

#### Subtheme: Stress and personal problems

Some aspects related to the patients’ personal circumstances can decrease their motivation and therefore challenge their process of quitting smoking. This could include psychosocial reasons such as stress, feeling lonely and depression.

“I told you the main reason, the pressure, the person’s circumstances… , we live for a long period outside our home country and tell ourselves that we should stay strong…. …” P8.

#### Subtheme: Cultural barriers for quitting

Few cultural barriers might have led to unsuccessful attempts of quitting smoking for some patients. These included considering smoking as a daily habit, surrounding community or environment of smokers, and perceiving smoking as a prestige by teenage smokers.

“And sometimes the reason behind me smoking during my medication period was my friends because when I was staying alone I wouldn’t smoke…. But when my friends are with me they would offer me a cigarette to smoke” P2.

### Theme 5: Patient-related facilitators for quitting smoking

#### Subtheme: Development of smoking related complications

Development of smoking related complications and fear of death were considered facilitators for quitting by both pharmacist and patient interviewees.

“age is a big factor. Because when they reach their 40s, smokers start getting health problems I mean smoking-related health problems … Then they start thinking about quitting… and they start thinking about their children and they want to be role models for them” PD1-01.

#### Subtheme: High motivation to quit

Patient motivation to quit was another facilitator for quitting as perceived by pharmacist and patient interviewees.

“from my experience, like I was really surprised with the number of patients or smokers who came and visited me to quit. They only saw the ad for the project and came to see me up to 70 % of those who quitted in my clinic were very willing to quit. Their motivation to quit facilitated their quitting” PD3-01.

#### Subtheme: Patients’ religious beliefs

Patients’ religious beliefs, in which some patients believed that God gave them the power to quit were considered facilitators for quitting by patients. Patients also deduced that their religious activities, like performing *Umrah* (lesser Hajj), and the holy month of Ramadan motivated them to stop smoking.

“I stopped for a while and then I started smoking again, but I have not been smoking for 3 days because I am going to Mecca inshallah to do Umrah, so I would like to stop smoking inshallah” P5.

#### Subtheme: External support

Patients indicated that the support they obtained from the surrounding people contributed to the success of their quitting. This support included family and friends’ emotional support and the support they obtained from other healthcare professionals.

“also my friend helped me… because we were quitting smoking together, so we were having a challenge, and I wanted to win the challenge! Also, my wife used to encourage me and calm me down whenever I was angry” P21.

### Theme 6: Program-related facilitators for quitting smoking

#### Subtheme: Smoking cessation medications

The patients reported that administration of smoking cessation medications, mainly NRT, assisted patients to some extent to successfully quit smoking.

“first of all, the drugs helped as they contain nicotine… I did not feel the taste of cigarettes, I used to smoke a cigarette, and then threw it…” P3.

#### Subtheme: Value of behavioral therapy in smoking cessation

Pharmacists considered that the behavioral therapy that they offered to patients was a facilitator for quitting. Pharmacists used coping and stress management strategies to help smokers identify any triggers for smoking and to engage in coping strategies to overcome these triggers.

Many of the pharmacists interviewed discussed the importance of showing emotional support and positive encouragement to smokers to help them change their behavior including engaging family members in the quitting process and communicating their belief in the patient’s ability and willingness to quit.

“I asked some patients if their spouses can come with them to the clinic, some spouses helped a lot. One spouse wanted to get pregnant she didn’t want to expose the baby to passive smoking so she came with the patient and really supported him in quitting” PD3-01.

### Theme 7: Internal strategies for program improvement

#### Subtheme: Recruiting motivated pharmacists and improving their training

Pharmacists suggested recruiting a higher number of motivated pharmacists. They also proposed improving the smoking cessation training program that they have received to focus more on behavioral therapy and on how to tailor the counseling to each patient individually.

“I suggest that the training workshop would include more simulation on what we are going to see with real patients for example you can include real smokers in the training. We can do role plays and you as trainers you can provide us feedback on our performance….” PD2-02.

#### Subtheme: Improving implementation of the program

Pharmacists proposed multiple strategies that would facilitate the program implementation. These included reducing the length of the questionnaires used for assessment, unifying the phone numbers used for follow-up calls, increasing duration of the program, improving premises such as providing private counseling rooms, increasing frequency of patient assessments and adding more smoking cessation medications such as varenicline to the program.

Furthermore, the patients suggested providing counseling over the phone, selecting appointment times that suit patients, offering group meetings to allow sharing experiences between smokers, ensuring willingness of smokers to quit before enrollment, and involving family as part of the quitting process.

“I have a suggestion, I do not mean to criticize, but involving family members in the program would help… as a smoker, having family members attend the sessions would help with increasing awareness, support and motivation” P17.

Both pharmacists and patients presumed that collaboration between pharmacists and physicians would improve the program.

“Maybe we can arrange with the physician himself or herself… we can have a collaborative model where pharmacists and physicians work together on helping smokers to quit” PD1-03.

#### Subtheme: Improving patient recruitment and follow-up in the program

The interviewees discussed several strategies to motivate smokers to join the program and to attend their follow-up sessions. These included promoting the program through media advertisements and offering incentives for patients who attend their follow-up sessions.

“Of course marketing in the first place… If there was an advertisement of any kind, as I saw there was at the entrance of the center. So I think if it was on the radio while people are going to their jobs in the morning they would be listening to the news or the Quran channel so if there was a small advertisement like the ones about health care I think it will encourage people, at the same time printed advertisements could be distributed to the patients at hospitals during their visits” P2.

## Discussion

To the best of our knowledge, this study is the first in Qatar and in the Middle East to qualitatively explore the experiences and perceptions of patients and pharmacists about a pharmacist-led smoking cessation program. In general, the study showed that both pharmacists and patients had positive views and experiences about the program. In addition, patients acknowledged the exceptional efforts that pharmacists exerted to help them in quitting smoking. These findings are in alignment with the published literature related to pharmacist role in smoking cessation interventions [25, 26]. It is promising to see the public recognition of the role of pharmacists in combating one of the major public health burdens in Qatar. National efforts to integrate pharmacists in smoking cessation programs in line with Qatar National Public Health Strategy 2017–2022 should be implemented [27].

Pharmacists discussed several challenges during the program’s implementation. For instance, they faced difficulties in initiating discussions with unmotivated patients. Establishing rapport and initiating discussions with smokers is an essential step in the process of smoking cessation [28]. Therefore, any proposed training offered to Qatar pharmacists in the future should focus on how to motivate smokers to quit including how to use motivational interviewing in smoking cessation. Moreover, the interviewed pharmacists identified lack of private consultation areas as a workplace barrier for the implementation of the program. A systematic review noted that around two-thirds of surveyed pharmacists in Canada agreed that a private area in the pharmacy was important to facilitate smoking cessation interventions and that 43 % of pharmacists in a study in Thailand believed that the pharmacy layout was an obstacle to providing counseling on smoking cessation [29]. To implement pharmacist-run smoking cessation programs, it is essential to include a designated private area for counseling in pharmacies to facilitate patient communication and interactions. Moreover, pharmacists reported lack of time as one of the challenges that they experienced when following-up patients. This is consistent with the findings of previous studies where pharmacists reported lack of time, understaffing, and busy schedules as barriers to providing smoking cessation services [29–32].

Pharmacists and patients stated several facilitators for the smoking cessation program implementation. One facilitator was the supportive management in their healthcare centers. This result is contradictory to the findings of some studies conducted outside Qatar where lack of management and organizational support were perceived as barriers to implementing smoking cessation programs [16, 33]. This reflects the huge efforts that the government of Qatar has put in place to reduce the burden of tobacco use in the country. Similarly, pharmacists proposed several strategies to improve the implementation of the program. One strategy was intensifying the training sessions delivered to pharmacists before implementing the program. Previous studies have demonstrated the benefits of intensive smoking cessation training programs on pharmacists and pharmacy students’ tobacco-related knowledge, self-efficacy, attitudes, and skills [34–38]. These findings imply that it is imperative to develop and implement intensive and comprehensive smoking cessation training programs for pharmacists in Qatar to improve the effectiveness of their counseling skills and ultimately reduce the burden of tobacco use in the country. Furthermore, the pharmacists proposed collaborating with other healthcare professionals in order to facilitate effective delivery of the program. Several studies suggested that involving multiple healthcare professionals as part of smoking cessation programs increases the effectiveness of these programs [33, 39–41]. Therefore, involving physicians and nurses in a collaborative approach would potentially be helpful in improving the effectiveness of pharmacist-run smoking cessation programs in future.

There are few limitations for the study. The number of pharmacists and patients who participated in the study may be considered low; however, the depth of collected information and achieving data saturation overcomes this limitation. Moreover, the study was conducted among primary care patients and pharmacists who participated in the program. Hence, study results may not be applied to other pharmacy or patient settings.

## Conclusions

In conclusion, the study participants considered that the pharmacist-provided tobacco cessation program was beneficial and contributed to advancing the role of the pharmacist in Qatar. Some perceived challenges to implementing the program were: time barriers, lack of patient commitment, cultural barriers, and lack of space. Conversely, management support, availability of consultation rooms, and other factors were identified as facilitators. There were many suggestions to improve the program, such as improving the training workshop, and increasing the program publicity. These findings can inform the development of successful pharmacist-led smoking cessation programs in Qatar and elsewhere.
